# Machine-learning-based prediction model of type 2 diabetes using liver enzymes: a cross-sectional study

**DOI:** 10.3389/fendo.2026.1905144

**Published:** 2026-09-03

**Authors:** Yaru Bi, Xiaojie Yuan, Yufeng Wang, Chenglin Sun, Suyan Tian

**Affiliations:** 1 Department of Endocrinology and Metabolism, First Hospital of Jilin University, Changchun, China; 2 Department of Clinical Nutrition, First Hospital of Jilin University, Changchun, China; 3 Department of Clinical Medicine, First Hospital of Jilin University, Changchun, China; 4 Division of Clinical Research, First Hospital of Jilin University, Changchun, China

**Keywords:** liver enzymes, type 2 diabetes, machine learning, prediction model, NHANES

## Abstract

**Introduction:**

Although observational studies have established associations between three major liver enzymes (alanine aminotransferase (ALT), aspartate aminotransferase (AST), and gamma-glutamyl transferase (GGT)) and type 2 diabetes (T2D), their ability to improve T2D identification using machine learning (ML) approaches remains underexplored. This study aimed to develop and validate ML-based diagnostic models for identifying T2D by incorporating these liver enzymes.

**Methods:**

Data from two independent cohorts were analyzed: the US National Health and Nutrition Examination Survey (N = 15,528) and a Chinese health examination database (N = 4,952). Twelve demographic and biochemical features were combined with liver enzymes to train eight ML models, including logistic regression, support vector machine, random forest, K-nearest neighbors, classification and regression trees, gradient boosting decision tree, LightGBM, and XGBoost. Model performance was assessed using standard metrics including accuracy, precision, recall, F1-score, and area under the curve (AUC).

**Results:**

Incorporating liver enzymes consistently improved model performance across the algorithms. The XGBoost model showed particularly strong performance, with baseline metrics (accuracy = 0.696, precision = 0.314, F1 = 0.450, AUC = 0.803) increasing to 0.743, 0.346, 0.468, and 0.814, respectively, after inclusion of liver enzymes. Comparable improvements were observed across algorithms and in both the US and Chinese cohorts.

**Conclusions:**

ML models incorporating routinely measured liver enzymes improve T2D identification across US and Chinese datasets. These findings indicate the potential utility of liver enzymes as accessible adjunctive indicators for diabetes risk stratification and improved T2D identification.

## Introduction

Diabetes mellitus is a chronic metabolic disorder characterized by insulin resistance and beta-cell dysfunction and represents a major global health challenge. Current estimates indicate that more over 10% of adults worldwide had diabetes in 2021, with projections suggesting an increase to 783 million cases by 2045 (1). Notably, type 2 diabetes (T2D) represents 90% of all diabetes cases. This condition markedly increases the risk of severe complications including cardiovascular disease, renal failure, vision impairment, lower-extremity amputations, and premature mortality (2, 3). The economic burden is also substantial, with global diabetes-related expenditures projected to reach $1.054 trillion by 2045 (1). These concerning trends underscore the urgent need for early identification of T2D.

Machine learning (ML), a core component of artificial intelligence, uses algorithmic approaches to extract meaningful patterns from complex datasets for predictive modeling (4). Recent advances in ML methods have transformed disease prediction, including T2D, by enabling early risk stratification and progression monitoring (5, 6). Existing T2D prediction models have incorporated diverse data types, including demographic characteristics such as sex, age, and ethnicity; lifestyle factors such as physical activity and smoking status; and basic clinical parameters (7). More sophisticated models have integrated biochemical markers such as fasting glucose and lipid profiles (8–11), genetic predisposition assessed using polygenic risk scores (12), and environmental exposure data such as chemical contaminants and heavy metals (13, 14).

Growing epidemiological evidence indicates a potential association between liver enzymes, particularly alanine aminotransferase (ALT), aspartate aminotransferase (AST), and gamma-glutamyl transferase (GGT), and T2D development (15–20). A recent cross-sectional analysis of the Azar cohort study (N = 14,865 participants aged 35–70 years) found significantly higher serum ALT, AST, and GGT levels in prediabetic and diabetic patients than in controls (*P* < 0.05). Multivariable logistic regression further revealed a dose-response relationship for all liver enzymes.

Although machine learning models achieve excellent T2D prediction, liver enzymes (ALT, AST, GGT) have not been fully leveraged despite their metabolic importance. We address this gap by developing and comparing multiple ML algorithms, including logistic regression (LR), support vector machines (SVM), K-nearest neighbors (KNN), classification and regression trees (CART), random forest (RF), gradient boosting decision trees (GBDT), LightGBM, and XGBoost, to quantify the incremental value of liver enzymes beyond established clinical features. The second aim of this study is to improve early screening approaches for T2D.

## Materials and methods

### Experimental data

This study analyzed two distinct datasets: (1) the US National Health and Nutrition Examination Survey (NHANES) spanning 2011–2018 and (2) a health examination study from Northeast China. The NHANES protocol was approved by the NCHS Ethics Review Board, and the Chinese protocol was approved by the Ethics Committee of the First Hospital of Jilin University (AF–IRB–032–06). All participants provided their written informed consents to participate in this study.

NHANES Cohort: We applied selection criteria consistent with our prior research (17). From the NHANES demographic, anthropometric, questionnaire, and biochemical data, we excluded participants aged <20 years; individuals with missing liver enzyme measurements; patients with hepatitis B/C; pregnant women; cancer patients; heavy alcohol consumers (>30 g/day [men] or >20 g/day [women]); and those diagnosed with diabetes before age 30 (to reduce potential misclassification of type 1 diabetes). The final analytical sample included 15,528 eligible participants.

In this cohort, non-alcoholic fatty liver disease (NAFLD) was defined using the Fatty Liver Index (FLI≥60), calculated from waist circumference (WC), triglycerides (TG), body mass index (BMI), and GGT. Diabetes was defined by meeting any of the following criteria: fasting plasma glucose (FPG) ≥7.0 mmol/L, 2-hour oral glucose tolerance test (OGTT) glucose ≥11.1 mmol/L, glycated hemoglobin (HbA1c) ≥6.5%, or current use of hypoglycemic medication or insulin.

Chinese Cohort: We recruited 5,300 adults (≥20 years) undergoing routine health examinations at the First Hospital of Jilin University (November 2022–March 2023). Demographic characteristics, anthropometric measurements, laboratory test results, and abdominal ultrasound data (for fatty liver diagnosis) were collected. Exclusion criteria included: non-T2D diagnoses; excessive alcohol intake (>30 g/day [men] or >20 g/day [women]); missing liver enzyme data; significant liver injury (ALT>250 IU/L, AST>200 IU/L, GGT>300 IU/L); and renal insufficiency (serum creatinine >333 μmol/L). After exclusions, 4,952 participants remained for analysis. In this cohort, T2D was defined based on FPG ≥7.0 mmol/L, HbA1c ≥6.5%, self-reported physician-diagnosed T2D, or current use of hypoglycemic medication or insulin.

### Data pre-processing

Before model development, several pre-processing steps were performed. Features with >15% missing data, including smoking status and low-density lipoprotein cholesterol, were excluded from further analysis. Features with ≤15% missingness, such as blood pressure, high-density lipoprotein cholesterol (HDL-C), and drinking status, were imputed using multiple imputation by chained equations (MICE) with 10 iterations. In addition, binary features, including sex (female/male), drinking status (no/yes), and NAFLD (no/yes), were encoded as 0/1 numeric variables, whereas continuous variables were standardized using the Z-score normalization. After pre-processing, 12 features were included for model development: sex, age, BMI, WC, systolic blood pressure (SBP), diastolic blood pressure (DBP), drinking status, serum creatinine (Scr), total cholesterol (TC), TG, HDL-C, NAFLD, and the three liver enzymes (ALT, AST, GGT).

### Model construction and evaluation

**Figure 1:**
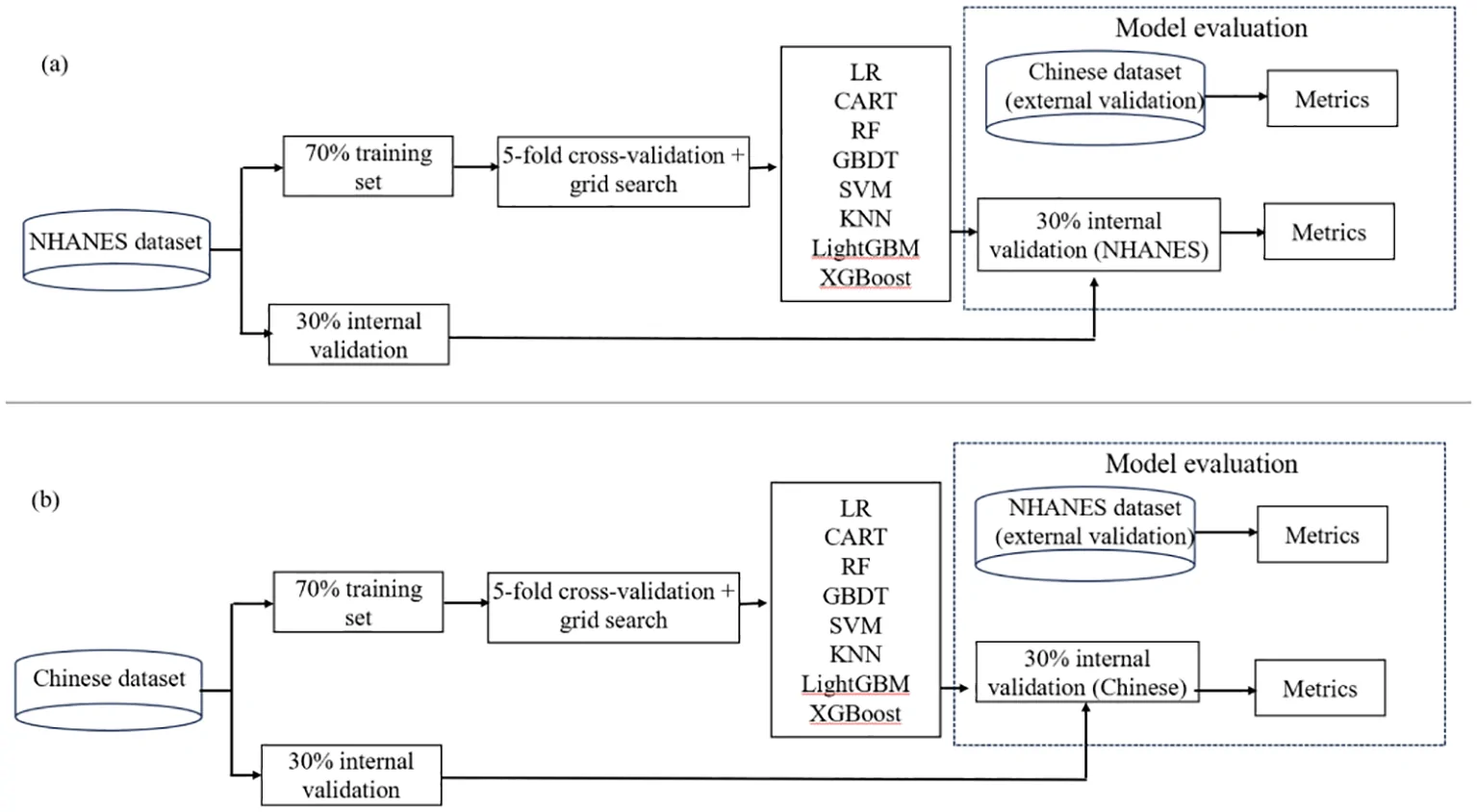
Flowchart of the machine learning model development and validation. **(a)** The NHANES dataset used as the training set, and the Chinese dataset as the external validation set; **(b)** the Chinese dataset used as the training set, and the NHANES dataset as the external validation set.

First, the NHANES dataset was divided into a training set and an internal validation set at a 7:3 ratio. A grid search strategy was combined with five-fold cross-validation within the training set to identify the optimal hyperparameters for each algorithm, while the Chinese dataset was used for external validation. The analytical strategy was then reversed: models were trained on the Chinese dataset and externally validated using NHANES data to assess model generalizability (**Figure 1**).

Eight ML algorithms were evaluated: logistic regression (LR), support vector machine (SVM), k-nearest neighbors (KNN), classification and regression trees (CART), random forest (RF), gradient-boosted decision trees (GBDT), LightGBM, and XGBoost. LR is a generalized linear model that uses the sigmoid function to convert linear risk scores into probabilities (ranging from 0 to 1) for event prediction. SVM determines an optimal hyperplane that maximizes the margin between distinct classes, allowing effective classification. KNN is a distance-based algorithm that classifies new samples based on the majority class among their *k* nearest neighbors. CART is a nonparametric decision tree method that recursively partitions data into binary branches to produce classification rules.

RF is as an ensemble model that constructs multiple decision trees trained on random subsets of features and samples, with final predictions obtained through majority voting. GBDT is a boosting method that sequentially trains weak learners by reweighting misclassified samples to enhance model performance. XGBoost and LightGBM are advanced, computationally efficient derivatives of GBDT. XGBoost improves computational speed and predictive accuracy, whereas LightGBM optimizes memory use and reduces training time. The hyperparameters explored are listed in **Supplementary Table 1**.

Model performance was evaluated using accuracy, precision, recall, F1-score, and the area under the receiver operating characteristic curve (AUC), with AUC prioritized as the primary indicator of predictive performance. Calibration was additionally evaluated using calibration curves, the calibration intercept and slope, and the Brier score (21). Decision curve analysis (DCA) was used to assess the clinical applicability and net benefit of the predictive models across different threshold probabilities (22).

To improve interpretability, we applied Shapley Additive exPlanations (SHAP) to the optimal model. SHAP quantifies the contribution of individual features to model predictions and reveals both the magnitude and direction (positive or negative) of their influence. Features with larger SHAP values were considered more influential in the model’s decision-making process.

### Comparison with risk score

We compared our models with the Chinese Diabetes Risk Score (CDRS) (23), which included six variables: age, sex, BMI, WC, SBP, and family history of diabetes. Because family history of diabetes was unavailable in the Chinese cohort, we used a modified CDRS that excluded this variable. The optimal cutoff for the modified CDRS was identified using the Youden index.

### Programming language

All ML models were implemented in Python (version 3.13.0). The XGBoost and LightGBM models were developed using the “xgboost” (version 3.3.0) and “lightgbm” (version 4.7.0) packages, respectively, while the other algorithms were built using “scikit-learn” (version 1.5.2). SHAP analysis was conducted with the “shap” (version 0.52.0) package. Fixed random seed (2026) was set for model training and cross-validation to ensure reproducibility of the results.

This study complies with the TRIPOD+AI reporting guideline (24), and the corresponding TRIPOD+AI checklist is provided as **Supplementary Material**.

## Results

### Baseline characteristics of the participants

The study included 15,528 participants from the NHANES dataset and 4,952 from the Chinese dataset, of whom 2,426 (15.62%) and 447 (9.03%) were identified as having T2D, respectively. Baseline characteristics of participants in both datasets are presented in **Supplementary Table 2**. Briefly, individuals with T2D had significantly higher values for age, BMI, WC, SBP, ALT, AST, and GGT than the non-diabetes group in both cohorts.

**Figure 2:**
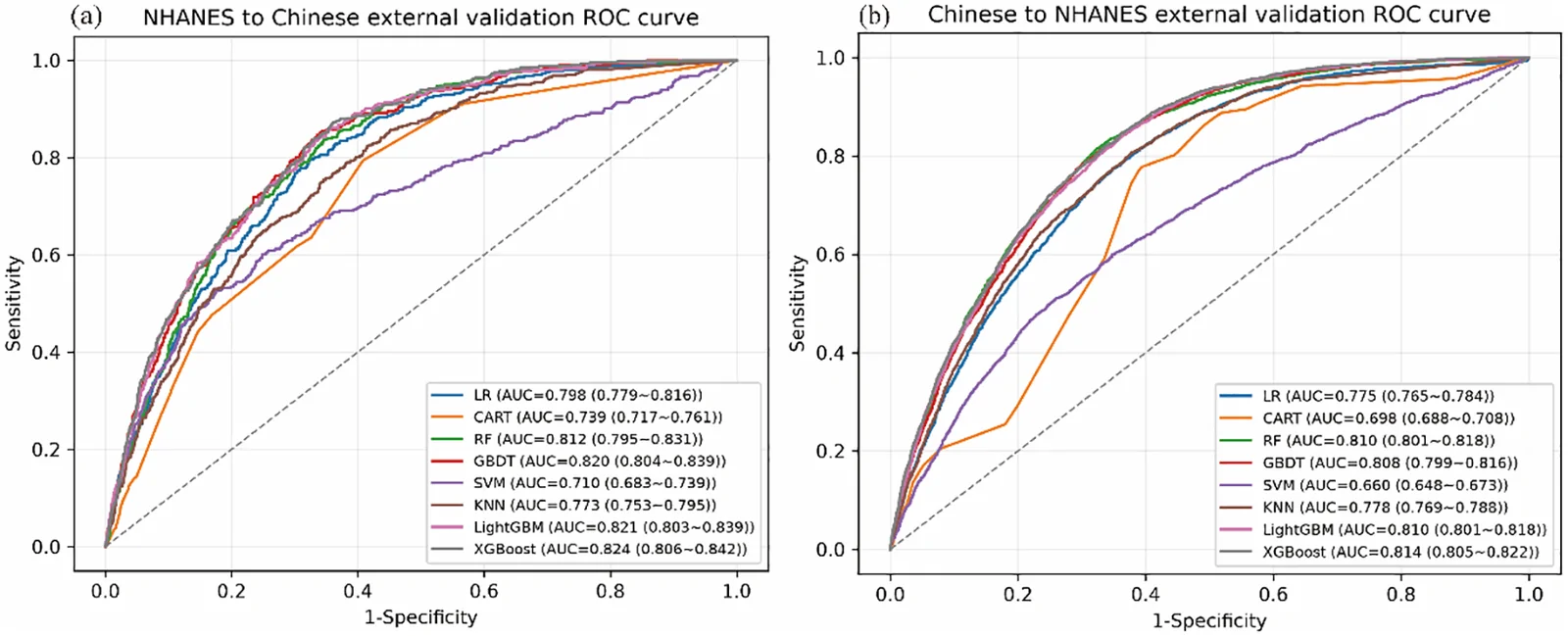
Receiver operating characteristic (ROC) curves of machine learning models from the Chinese external validation **(a)** and the NHANES external validation **(b)**.

The NHANES dataset served as the training set for developing eight ML models based on 12 features: sex, age, BMI, WC, SBP, DBP, NAFLD status, Scr, TC, TG, HDL-C, and drinking status. The performance of these models on the Chinese external validation set is presented in **Table 1**. In addition, the inclusion of ALT, AST, and GGT improved the performance of T2D detection. **Figure 2a** presents the receiver operating characteristic (ROC) curves of these models. DeLong’s test indicated that the AUC improvements were statistically significant (*P* < 0.05) except for CART and KNN. The XGBoost model yielded the best performance. Without liver enzymes, the XGBoost model achieved the following metrics: accuracy = 0.709, precision = 0.207, recall = 0.785, F1-score = 0.328, and AUC = 0.802. After inclusion of liver enzymes, these metrics changed to: accuracy = 0.707, precision = 0.207, recall = 0.794, F1-score = 0.328, and AUC = 0.824. **Supplementary Tables 3**, **4** provide the confusion matrices, specificity, positive predictive value, and negative predictive value for the XGBoost models in the Chinese external validation set.

**Table 1:** Performance metrics of the prediction models validated on the Chinese cohort.

Model	Accuracy	Precision	Recall	F1-score	AUC (95% CI)	DeLong’s test *P*
LR	0.790	0.231	0.570	0.329	0.792 (0.772, 0.811)	0.047
LR (+enzymes)	0.786 (−0.4%)	0.235 (+0.4%)	0.609 (+3.9%)	0.339 (+1%)	0.798 (0.779, 0.816)	
CART	0.565	0.154	0.855	0.262	0.736 (0.714, 0.758)	0.459
CART (+enzymes)	0.610 (+4.5%)	0.162 (+0.8%)	0.794 (−6.1%)	0.269 (+0.7%)	0.739 (0.717, 0.761)	
RF	0.751	0.220	0.689	0.333	0.800 (0.783, 0.819)	0.001
RF (+enzymes)	0.714 (−3.7%)	0.207 (−1.3%)	0.765 (+7.6%)	0.326 (−0.7%)	0.812 (0.795, 0.831)	
GBDT	0.758	0.225	0.689	0.339	0.802 (0.784, 0.821)	<0.001
GBDT (+enzymes)	0.741 (−1.7%)	0.220 (−0.5%)	0.736 (+4.7%)	0.339 (+0%)	0.820 (0.804, 0.839)	
SVM	0.733	0.163	0.472	0.242	0.638 (0.608, 0.669)	<0.001
SVM (+enzymes)	0.812 (+7.9%)	0.239 (+7.6%)	0.494 (+2.2%)	0.322 (+8%)	0.710 (0.683, 0.739)	
KNN	0.719	0.186	0.624	0.286	0.760 (0.741, 0.781)	0.106
KNN (+enzymes)	0.711 (−0.8%)	0.190 (+0.4%)	0.678 (+5.4%)	0.297 (+1.1%)	0.773 (0.753, 0.795)	
LightGBM	0.806	0.247	0.562	0.343	0.806 (0.788, 0.824)	<0.001
LightGBM (+enzymes)	0.704 (−10.2%)	0.205 (−4.2%)	0.787 (+22.5%)	0.325 (−1.8%)	0.821 (0.803, 0.839)	
XGBoost	0.709	0.207	0.785	0.328	0.802 (0.784, 0.820)	<0.001
XGBoost (+enzymes)	0.707 (−0.2%)	0.207 (+0%)	0.794 (+0.9%)	0.328 (+0%)	0.824 (0.806, 0.842)	

**Figure 3:**
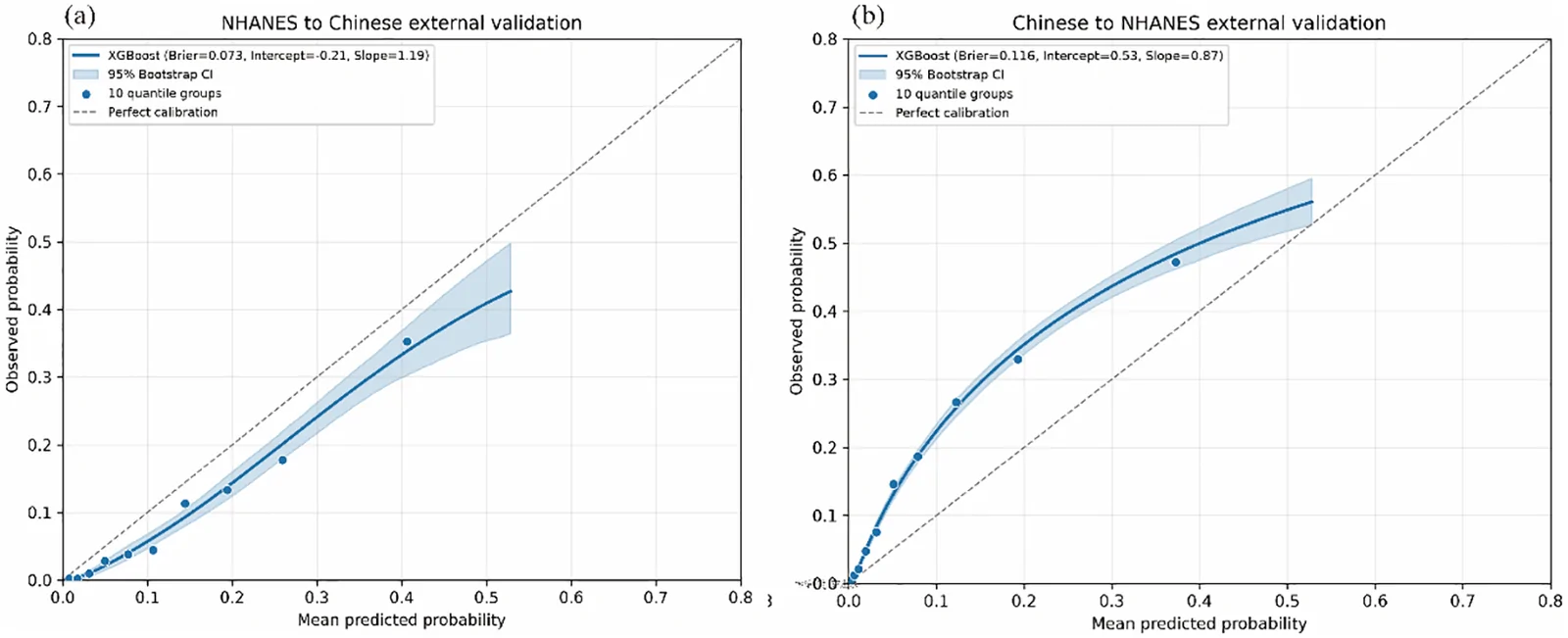
Calibration curve of the XGBoost models from the Chinese external validation **(a)** and the NHANES external validation **(b)**.

**Figure 4:**
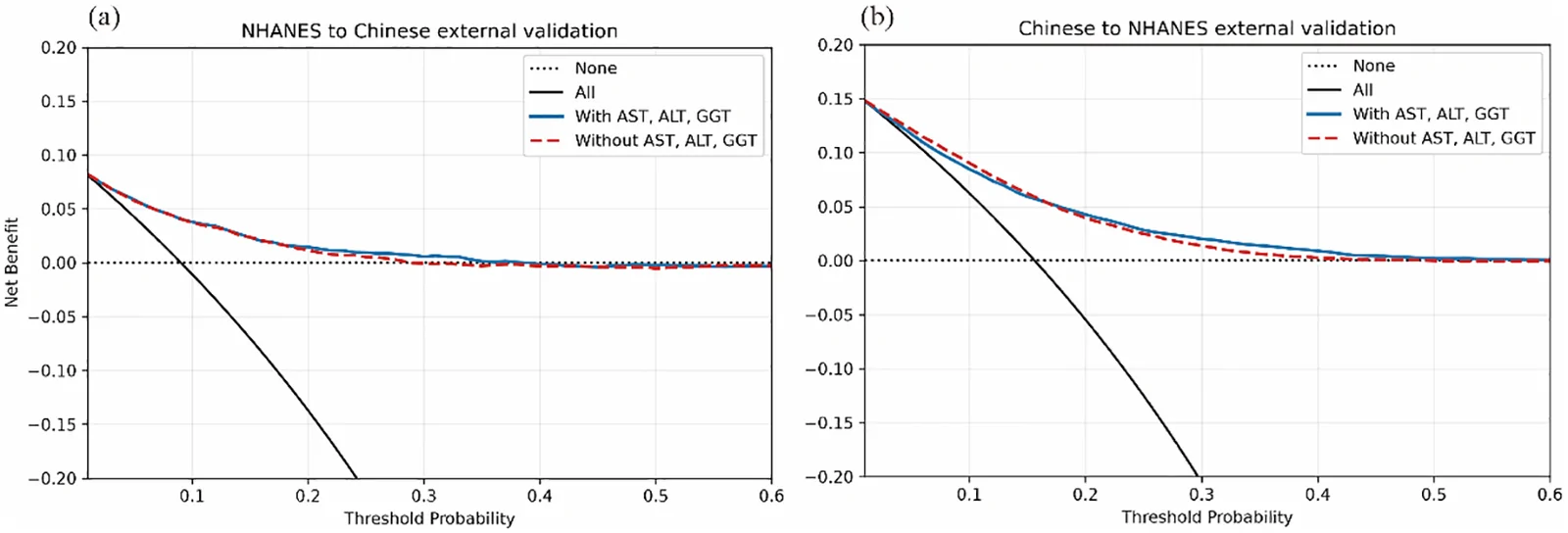
Decision curve analysis of the XGBoost models from the Chinese external validation **(a)** and the NHANES external validation **(b)**.

**Figure 5:**
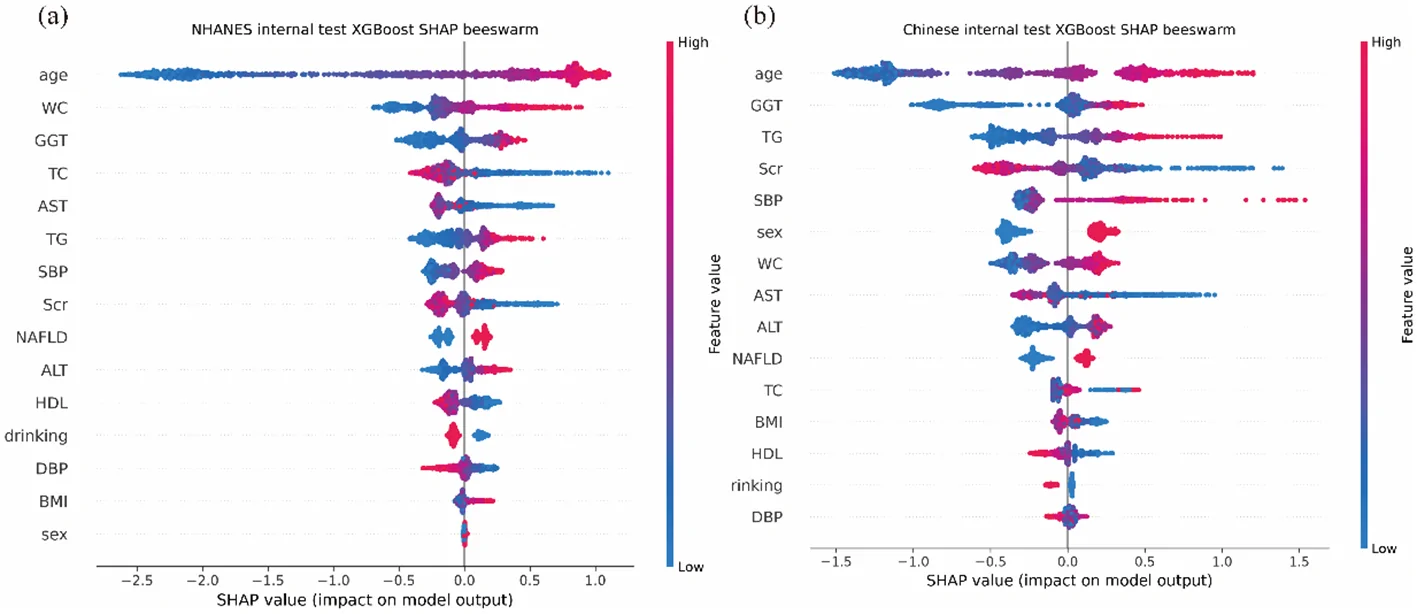
Shapley Additive exPlanations (SHAP) values for the XGBoost models features from the NHANES internal test **(a)** and Chinese internal test **(b)**.

For the XGBoost model, the calibration curve showed satisfactory calibration in the Chinese external validation set (Brier score: 0.07, calibration intercept: -0.21, calibration slope: 1.19) (**Figure 3a**). The DCA curve indicated that the XGBoost model achieved greater net benefit than the treat-all or treat-none strategies across a clinically meaningful range of threshold probabilities (0.1 to 0.35). Furthermore, the XGBoost model incorporating liver enzymes exhibited a slightly higher net benefit than the model without liver enzymes (threshold range: 0.2–0.35) (**Figure 4a**). SHAP analysis indicated that age had the largest predictive weight for T2D detection, followed by WC and GGT. Among the liver enzymes, GGT, AST, and ALT ranked 3rd, 5th, and 10th in predictive contribution, respectively. Within this model, liver enzymes showed a greater contribution to T2D detection than BMI, a well-established marker linked to T2D (**Figure 5a**).

Subsequently, eight machine learning models were trained on the Chinese dataset using twelve baseline features. After ALT, AST, and GGT were incorporated into the models, performance improved in the NHANES external validation set except for CART, and the improvements were statistically significant (DeLong’s test *P* < 0.05) (**Table 2**, **Figure 2b**). The performance gains were particularly evident for ensemble methods. For the GBDT model, consistent gains were observed across all evaluation metrics. Accuracy increased from 0.709 to 0.717 (+0.008), and precision rose from 0.316 to 0.328 (+0.012). The recall rate increased from 0.737 to 0.773 (+0.036), accompanied by an F1-score improvement from 0.442 to 0.461(+0.019). Most notably, AUC increased from 0.792 to 0.808 (+0.016), indicating enhanced discriminative ability.

**Table 2:** Performance metrics of the prediction models validated on the NHANES cohort.

Model	Accuracy	Precision	Recall	F1-score	AUC (95% CI)	DeLong’s test *P*
LR	0.743	0.326	0.602	0.423	0.767 (0.758, 0.777)	<0.001
LR (+enzymes)	0.762 (+1.9%)	0.340 (+1.4%)	0.559 (−4.3%)	0.423 (0%)	0.775 (0.765, 0.784)	
CART	0.562	0.244	0.859	0.380	0.709 (0.699, 0.719)	0.002
CART (+enzymes)	0.554 (−0.8%)	0.243 (−0.1%)	0.876 (+1.7%)	0.381 (+0.1%)	0.698 (0.688, 0.708)	
RF	0.631	0.279	0.863	0.422	0.794 (0.785, 0.803)	<0.001
RF (+enzymes)	0.667 (+3.6%)	0.300 (+2.1%)	0.849 (−1.4%)	0.443 (+2.1%)	0.810 (0.801, 0.818)	
GBDT	0.709	0.316	0.737	0.442	0.792 (0.783, 0.800)	<0.001
GBDT (+enzymes)	0.717 (+0.8%)	0.328 (+1.2%)	0.773 (+3.6%)	0.461 (+1.9%)	0.808 (0.799, 0.816)	
SVM	0.641	0.237	0.584	0.337	0.651 (0.640, 0.662)	0.090
SVM (+enzymes)	0.649 (+0.8%)	0.242 (+0.5%)	0.586 (+0.2%)	0.343 (+0.6%)	0.660 (0.648, 0.673)	
KNN	0.619	0.270	0.840	0.408	0.771 (0.763, 0.780)	0.030
KNN (+enzymes)	0.607 (−1.2%)	0.265 (−0.5%)	0.853 (+1.3%)	0.404 (−0.4%)	0.778 (0.769, 0.788)	
LightGBM	0.678	0.300	0.799	0.437	0.798 (0.790, 0.806)	<0.001
LightGBM (+enzymes)	0.745 (+6.7%)	0.344 (+4.4%)	0.698 (−10.1%)	0.461 (+2.4%)	0.810 (0.801, 0.818)	
XGBoost	0.696	0.314	0.797	0.450	0.803 (0.794, 0.811)	<0.001
XGBoost (+enzymes)	0.743 (+4.7%)	0.346 (+3.2%)	0.723 (−7.4%)	0.468 (+1.8%)	0.814 (0.805, 0.822)	

The XGBoost model showed similar improvements. Accuracy increased from 0.696 to 0.743 (+0.047), while precision improved by 0.032 (0.314 to 0.346). A modest gain was observed for F1-score (0.450 to 0.468, + 0.018). The AUC increased from 0.803 to 0.814 (+0.011), and this improvement was statistically significance (DeLong’s test *P* < 0.001), maintaining XGBoost as the highest-performing model. **Supplementary Table 3** provides the confusion matrix obtained from the NHANES external validation cohort.

Calibration curves indicated moderate agreement (Brier score: 0.12, calibration intercept: 0.53, calibration slope: 0.87) (**Figure 3b**). The DCA curve for the XGBoost model incorporating liver enzymes lies above that of the model without liver enzymes (threshold range: 0.25–0.45), indicating greater net clinical benefit (**Figure 4b**). SHAP analyses identified the leading features in descending order of contribution as age, GGT, and TG. Among the liver enzymes, GGT, AST, and ALT ranked 2nd, 8th, and 9th, respectively (**Figure 5b**). Within this fitted model, liver enzymes had larger mean absolute SHAP values than BMI, a well-established correlate of T2D.

We compared model performance with the modified CDRS. In the Chinese external validation cohort, the modified CDRS achieved an AUC of 0.776 (95% CI: 0.756–0.795) at the optimal cutoff of 28.5, with a sensitivity of 80.3% and specificity of 63.3%. In comparison, our XGBoost model showed superior performance with an AUC of 0.824 (95% CI: 0.806–0.842). The DeLong’s test indicated a statistically significant difference between the two AUCs (*P* < 0.05). Similar findings were observed in the NHANES external validation cohort, where the modified CDRS achieved an AUC of 0.780 (95%CI: 0.771–0.788) compared with 0.814 (95% CI: 0.805–0.822) for our XGBoost model (*P* < 0.05) (**Supplementary Table 5**).

## Discussion

This study demonstrated that incorporating liver enzymes improved the performance of models for identifying T2D across different populations. The consistent improvements in model metrics and robust performance during external validation suggest that liver enzymes provide incremental predictive value beyond routine clinical features. This finding is particularly notable because liver enzymes maintained their predictive power even after adjustment for established clinical correlates of T2D, including BMI, WC, and lipid profiles.

Incorporating liver enzymes improved model predictive performance for T2D identification and conferred a favorable net benefit across clinically relevant threshold probabilities. Although inclusion of liver enzymes slightly increased model complexity, these routinely measured, low-cost laboratory tests impose no additional clinical or economic burden. Furthermore, liver enzymes are nonspecific markers influenced by obesity and hepatic steatosis. During model construction, conventional metabolic factors and fatty liver were already included in the models. The improved predictive capacity suggests that liver enzymes provide additional information beyond existing metabolic factors and hepatic steatosis.

The superior performance of XGBoost among the eight ML algorithms evaluated is consistent with previous studies showing the effectiveness of ensemble-based methods for medical prediction tasks (25–27). Our SHAP analysis identified several clinically meaningful patterns. Among the three liver enzymes, GGT made the most prominent contribution to model prediction, with its SHAP value exceeding those of conventional metabolic markers such as blood pressure. All three enzymes also showed substantial absolute SHAP values that were larger than those of BMI in the models. The predictive contribution of liver enzymes was consistent across both US and Chinese datasets, suggesting that these biomarkers may be generalizable across ethnicities.

The association of GGT with T2D is explained by its glutathione-related roles in oxidative stress-mediated beta-cell damage, inflammation-induced insulin resistance, and dysregulation of hepatic glucose metabolism (28–30). ALT elevations are associated with liver fat content and hepatic insulin resistance (31, 32). These enzymes collectively indicate progressive metabolic liver injury, with GGT reflecting redox imbalance, ALT indicating steatotic lipotoxicity, and AST signaling mitochondrial distress during NASH progression (28, 31, 33). Their complementary elevations capture distinct diabetes-pathogenic processes, from initial hepatic lipid accumulation (ALT) to subsequent inflammatory/oxidative damage (GGT/AST), explaining their combined incremental value beyond conventional risk factors.

Although the current study leveraged two large, well-characterized datasets, several limitations warrant consideration. First, inclusion of FLI-defined NAFLD status alongside its constituent indicators (BMI, WC, and TG) may introduce construct overlap, particularly because GGT, a component of FLI, is subsequently added to the model to evaluate its incremental value. Nevertheless, we used binary FLI-based NAFLD status rather than continuous FLI scores, meaning that the continuous, graded information remains available for the model to leverage. Second, the diagnostic criteria for NAFLD differed between the cohorts, potentially introducing minor bias. Third, the prevalence of NAFLD was 47.05% and 51.80% in the NHANES and Chinese cohorts, which was higher than the reported NAFLD prevalence of approximately 32% in the general adult population (34). The generalizability to low-risk community settings therefore requires further validation. Lastly, the inherently observational design precludes definitive causal inference regarding the observed association.

## Conclusions

In conclusion, this study provides consistent evidence that incorporating liver enzymes could improve the performance of ML-based diagnostic prediction models for T2D across different populations and algorithms. These findings indicate that routinely available liver enzymes could improve the identification of individuals with T2D. Future longitudinal research should examine the temporal relationship between liver enzyme changes and diabetes development, elucidate the underlying mechanisms linking these changes to diabetes pathogenesis, and explore the potential for personalized prevention strategies based on these biomarkers.

## Data Availability

The original contributions presented in the study are included in the article/**Supplementary Material**. Further inquiries can be directed to the corresponding authors.
