# Supplementary material for: Machine-learning-based prediction model of type 2 diabetes using liver enzymes: a cross-sectional study
Source: Front Endocrinol (Lausanne). 2026 Sep 3;17:1905144. doi: 10.3389/fendo.2026.1905144 (PMC13581821; doi:10.3389/fendo.2026.1905144)
Supplement: Supplementary file 1 [file DataSheet1.pdf]

Supplementary Table 1. Hyperparameter tuning for machine learning algorithms

Algorithms	Packages	Tuning parameters	Search range	Optimal value
LR	scikit-learn (1.5.2)	C	[0.01, 0.1, 1, 10, 100]	[1]
		l1_ratio	[1.0, 0.0]	[1.0]
CART	scikit-learn (1.5.2)	Criterion	["gini", "entropy","log_loss"]	[Gini]
		max_depth	[3, 4, 5, 6, 8, 10, 12, None]	[8]
		min_samples_split	[2, 5, 10, 20, 50]	[2]
		min_samples_leaf	[1, 2, 5, 10, 20, 50]	[20]
		max_leaf_nodes	[None, 8, 16, 32, 64]	[32]
		ccp_alpha	[0.0, 0.0001, 0.0005, 0.001, 0.005, 0.01]	[0.0]
RF	scikit-learn (1.5.2)	n_estimators	[300, 500]	[500]
		max_depth	[5, 10, 15, None]	[10]
		min_samples_split	[2, 5, 10]	[5]
		min_samples_leaf	[1, 2, 5]	[5]
		max_features	["sqrt", "log2", 0.5]	[0.5]
GBDT	scikit-learn (1.5.2)	n_estimators	[100, 200, 300],	[200]
		learning_rate	[0.01, 0.05, 0.1],	[0.1]
		max_depth	[2, 3, 4]	[2]
		min_samples_leaf	[1, 5, 10],	[10]
		subsample	[0.8, 1.0]	[1.0]
SVM	scikit-learn (1.5.2)	kernel	[rbf]	[rbf]
		C	[0.1, 0.3, 1, 3, 10]	[0.1]
		gamma	["scale", 0.003, 0.01, 0.03, 0.1, 0.3]	[0.01]
KNN	scikit-learn (1.5.2)	n_neighbors	[3, 5, 7, 9, 11, 15, 21, 31]	[31]
		weights	["uniform", "distance"]	[distance]
		metric:	["minkowski"]	["minkowski"]
		p	[1, 2]	[1]
		leaf_size	[20, 30, 40]	[30]
LightGBM	lightgbm (4.7.0)	n_estimators	[200, 500]	[200]
		learning_rate	[0.03, 0.1]	[0.03]
		num_leaves	[15, 31, 63]	[15]
		max_depth	[-1, 5, 10]	[-1]
		min_child_samples	[10, 20, 40]	[40]
		subsample	[0.8, 1.0]	[0.8]
		colsample_bytree	[0.8, 1.0]	[0.8]
XGBoost	xgboost (3.3.0)	n_estimators	[200, 300, 400, 600, 800]	[300]
		learning_rate	[0.02, 0.03, 0.05, 0.08, 0.1]	[0.03]
		max_depth	[2,3, 4, 5]	[3]
		min_child_weight	[1, 3, 5,10]	[3]
		subsample	[0.7, 0.8, 0.9, 1.0]	[0.8]
		colsample_bytree	[0.7, 0.8, 0.9, 1.0]	[0.8]
		gamma	[0, 0.05, 0.1, 0.2]	[0.2]
		reg_alpha	[0, 0.01, 0.1, 1.0]	[0]
		reg_lambda	[0.5, 1.0, 2.0, 5.0, 10.0]	[10]

Supplementary Table 2 Characteristics of the participants in the two datasets

Variables	NHANES dataset			Chinese dataset		
	Non-T2D (N=13102)	T2D (N=2426)	*P*	Non-T2D (N=4505)	T2D (N=447)	*P*
Male, N (%)	6280 (47.93)	1247 (51.40)	0.002	2159 (47.92)	302 (67.56)	<0.001
Age, (age)	45.37±16.18	59.64±12.46	<0.001	46.29±12.57	55.83±10.76	<0.001
Drinking (yes), N (%)	8085 (61.71)	1138 (46.91)	<0.001	985 (21.86)	76 (17.00)	0.017
BMI (kg/m^2)	28.69±6.69	32.10±7.32	<0.001	25.25±3.72	26.92±3.47	<0.001
WC (cm)	97.47±15.93	108.40±16.18	<0.001	84.78±10.72	91.58±8.88	<0.001
SBP (mmHg)	121.75±16.76	131.65±18.40	<0.001	129.76±17.59	143.27±20.69	<0.001
DBP (mmHg)	71.08±11.67	70.31±13.57	0.013	143.27±20.70	84.11±12.13	<0.001
ALT (IU/L)	20 (15, 27)	22 (16, 31)	<0.001	20 (14.4, 30)	25.3 (18.5, 37.0)	<0.001
AST (IU/L)	22 (18, 26)	22 (18, 28)	0.014	21.9 (18.5, 26.8)	23.2 (19.1, 30.3)	<0.001
GGT (IU/L)	19 (14, 28)	24 (17, 38)	<0.001	24.2 (16.5, 38.7)	35.1 (25.1, 56.1)	<0.001
TC (mmol/l)	4.99±1.02	4.86±1.24	<0.001	5.25±1.02	5.57±1.24	<0.001
TG (mmol/l)	1.62±1.24	1.71 (1.17, 2.55)		1.25 (0.89, 1.84)	1.87 (1.30, 2.81)	<0.001
HDL-C (mmol/L)	1.39±0.41	1.26±0.38	<0.001	1.37±0.31	1.27±0.27	<0.001
NAFLD (%)	5583 (42.61)	1723 (71.02)	<0.001	2206 (48.97)	359 (80.31)	<0.001

Note: Data were expressed as the mean±standard deviation (normal distribution) or median (P25, P75) (non-normal distribution) for continuous variables, and N (percentages) for categorical variables.

Supplementary Table 3 Confusion matrix of the XGBoost model on Chinese external validation

**XGBoost (+enzymes) NHANES to Chinese**	Predicted negative	Predicted positive
Actual negative	TN = 3145	FP = 1360
Actual positive	FN = 92	TP = 355
**XGBoost (+enzymes) Chinese to NHANES**		
Actual negative	TN = 9783	FP = 3319
Actual positive	FN = 672	TP = 1754

Note: TN: true negative; FP: false positive; FN: false negative; TP: true positive

Supplementary Table 4 Sensitivity, specificity, PPV, NPV, PR-AUC of the final XGBoost models (with liver enzymes) in the external validation set

	Sensitivity	specificity	PPV	NPV	PR-AUC
Chinese set (external)	0.794	0.698	0.207	0.972	0.264
NHANES set (external)	0.723	0.747	0.346	0.936	0.359

Note: PPV: positive predictive value, NPV: negative predictive value; PR-AUC: Precision-Recall Curve

Supplementary Table 5 Performance comparison of the modified-Chinese Diabetes Risk Score (CDRS) and XGBoost models

	**Accuracy**	**Precision**	**Sensitivity**	**F1**	**Specificity**	**AUC (95%CI)**	**DeLong's test**
**Modified-CDRS (Chinese external validation)**	0.648	0.178	0.803	0.292	0.633	0.776 (0.756-0.795)	<0.001
**XGBoost (Chinese external validation)**	0.707	0.207	0.794	0.328	0.698	0.824 (0.806,0.842)	
**Modified-CDRS (NHANES external validation)**	0.630	0.278	0.854	0.419	0.588	0.780 (0.771, 0.788)	<0.001
**XGBoost (NHANES external validation)**	0.743	0.346	0.723	0.468	0.747	0.814 (0.805,0.822)	
